# Evaluation of the immunogenicity and protective effects of a trivalent chimeric norovirus P particle immunogen displaying influenza HA2 from subtypes H1, H3 and B

**DOI:** 10.1038/emi.2016.51

**Published:** 2016-05-25

**Authors:** Xin Gong, He Yin, Yuhua Shi, Xiaoqiu He, Yongjiao Yu, Shanshan Guan, Ziyu Kuai, Nasteha M Haji, Nafisa M Haji, Wei Kong, Yaming Shan

**Affiliations:** 1 National Engineering Laboratory for AIDS Vaccine, School of Life Sciences, Jilin University, Changchun 130012, Jilin Province, China; 2 Norman Bethune Health Science Center, Jilin University, Changchun 130021, Jilin Province, China; 3 Key Laboratory for Molecular Enzymology and Engineering, The Ministry of Education, School of Life Sciences, Jilin University, Changchun 130012, Jilin Province, China

**Keywords:** influenza virus, hemagglutinin 2, trivalent chimeric immunogen, norovirus P particle, protective effects

## Abstract

The ectodomain of the influenza A virus (IAV) hemagglutinin (HA) stem is highly conserved across strains and has shown promise as a universal influenza vaccine in a mouse model. In this study, potential B-cell epitopes were found through sequence alignment and epitope prediction in a stem fragment, HA2:90-105, which is highly conserved among virus subtypes H1, H3 and B. A norovirus (NoV) P particle platform was used to express the HA2:90-105 sequences from subtypes H1, H3 and B in loops 1, 2 and 3 of the protrusion (P) domain, respectively. Through mouse immunization and microneutralization assays, the immunogenicity and protective efficacy of the chimeric NoV P particle (trivalent HA2-PP) were tested against infection with three subtypes (H1N1, H3N2 and B) of IAV in Madin–Darby canine kidney cells. The protective efficacy of the trivalent HA2-PP was also evaluated preliminarily *in vivo* by virus challenge in the mouse model. The trivalent HA2-PP immunogen induced significant IgG antibody responses, which could be enhanced by a virus booster vaccination. Moreover, the trivalent HA2-PP immunogen also demonstrated *in vitro* neutralization of the H3 and B viruses, and *in vivo* protection against the H3 virus. Our results support the notion that a broadly protective vaccine approach using an HA2-based NoV P particle platform can provide cross-protection against challenge viruses of different IAV subtypes. The efficacy of the immunogen should be further enhanced for practicality, and a better understanding of the protective immune mechanism will be critical for the development of HA2-based multivalent vaccines.

## INTRODUCTION

Influenza A virus (IAV) infections are a significant cause of morbidity and mortality in humans.^1^ Traditional flu vaccines offer protection against three different viruses that are expected to circulate throughout the influenza season. These strains include one type B and two type A (H1, H3 subtypes) strains. However, the traditionally administered trivalent influenza vaccines have a long manufacturing process,^2, 3, 4^ and the antigenic match between circulating viruses during a given influenza season is limited.^5^ Therefore, new approaches and strategies for the development of broadly protective vaccines are urgently needed.

Recent studies showed that conserved influenza viral proteins, including the hemagglutinin (HA) 2 subunit, matrix 2 and other structural proteins, can provide protective immune responses against heterosubtypic influenza viruses.^6^ HA, consisting of a globular HA1 domain and a more conserved HA2 stem, is the main envelope glycoprotein on the surface of IAV that binds with host cell receptors during infection,^7^ and it mediates subsequent endocytosis and membrane fusion.^8^ Because HA2 is partially hidden inside the virus and is poorly immunogenic, several approaches were developed to increase its immunogenicity, including fusion of the HA2 peptide to keyhole limpet hemocyanin (KLH),^9^ expression of the trimeric HA stem fragment ^10, 11^ and expression of influenza virus-like particles.^12^ These constructs have been shown to induce immune responses against HA, resulting in varying degrees of protection in different animal models against different challenge strains.

The norovirus (NoV) P particle, a subviral particle formed by 24 copies of the protrusion (P) domain of the NoV capsid protein, is easily expressed and purified, extremely stable and highly immunogenic.^13, 14^ Each P domain contains three surface loops, which have been demonstrated to be useful for foreign antigen presentation.^15^ In recent studies, IAV matrix 2 antigens were successfully inserted into a surface loop on the P domain of NoV, and immunization of mice with the chimeric P particles was shown to induce significant immune responses to the inserted antigen and to provide protection against viral challenge.^16, 17^ Thus, the P particle is considered an excellent platform for vaccine development against infectious diseases.

Recently, we identified a conserved stem fragment, HA2:90-105, located in the turning point of the HA neck that undergoes conformational change during membrane fusion, that induces strong humoral immune responses and broad neutralizing activity against group 2 viruses of the H3 subtype.^18^ In this study, based on a multi-subtype HA2 alignment and prediction of B-cell epitopes, a NoV P particle platform expressing 24 copies of trivalent HA2:90-105 on its surface loops was constructed. Furthermore, the immunogenicity and protective efficacy of this immunogen against challenge with three different subtypes of IAV were evaluated in both Madin–Darby canine kidney cells and a mouse model.

## MATERIALS AND METHODS

### Animals and ethics statement

BALB/c female mice, 6−8-week-old (18−20 g), were purchased from the Changchun Institute of Biological Products Co., Ltd (Changchun, China). The animal experiments in this study were carried out in accordance with the Regulations and the Administration of Affairs Concerning Experimental Animals approved by the State Council of People's Republic of China (11-14-1988). The Institutional Animal Care and Use Committee (IACUC) of Jilin University approved all animal procedures (permit number: SCXK 2013-0001).

### Cells and influenza viruses

Madin–Darby canine kidney cells and human adenocarcinoma (A549) cells were grown in a T-flask with Dulbecco's modified Eagle's medium and 10% fetal bovine serum at 37 °C in 5% CO_2_. A/17/California/2009/38(H1N1), A/17/Perth/16/2009(H3N2), A/Wisconsin/67/2005(H3N2), A/Texas/JMM_30/2012(H3N2), B/56/Brisbane/60/80 and B/60/Massachusetts/20/2012 viruses were obtained from Changchun BCHT Pharmaceutical Co., Ltd (Changchun, China).

### Sequence alignment and B-cell epitope prediction

For the alignment of HA fragments of the H1, H3 and B subtypes, a total of 17 524, 10 763 and 4022 HA2 sequences, respectively, was downloaded from the National Center for Biotechnology Information (NCBI) Influenza Virus Sequence Database^19^ and were analyzed using software according to a previously described procedure.^20, 21^ Sequence analysis was performed using ClustalW v1.4, which is included in BioEdit v7.0.0. Alignments were performed using MEGA version 5.0, which allowed the comprehensive analysis of accessibility, flexibility and hydrophilicity. The Kolaskar & Tongaonkar antigenicity method^22^ from the Immune Epitope Database (IEDB)^23^ was used to predict B-cell epitopes.

### Construction of a trivalent HA2-P particle (trivalent HA2-PP) chimeric immunogen

A trivalent HA2-PP chimeric protein immunogen was constructed by cloning the IAV consensus HA2:90-105 genes from the H1, H3 and B subtypes into a P particle expression vector (pET-28a (+) containing the P-domain sequence of NoV, genogroup II, cluster 4 (GII.4), with six His residues at the C-terminus; synthesized by Generay Biotechnology Corporation (Shanghai, China)). In brief, gene sequences were synthesized and cloned into the P particle expression vector to produce the immunogen as follows: one copy of the H1 IAV consensus HA2:90-105 peptide sequence (DIWTYNAELLVLLENE) was inserted between the G274 and T275 residues of loop 1, one copy of the H3 IAV consensus HA2:90-105 peptide sequence (DLWSYNAELLVALENQ) was inserted between the S372 and N373 residues of loop 2 and one copy of the B IAV consensus HA2:90-105 peptide sequence (DTISSQIELAVLLSNE) was inserted between the G392 and S393 residues of loop 3 of the NoV P domain via a GGGGS linker.

### Expression, purification and confirmation of the trivalent HA2-PP chimeric immunogen

The expression and purification of trivalent HA2-PP and wild-type (WT) P particles were performed according to established protocols^24^ with modifications. Inclusion bodies were incubated overnight in a lysis solution (8 M urea, 0.5 M NaCl, 20 mM imidazole, 5 mM β-mercaptoethanol in phosphate-buffered saline (PBS), pH 8.0) and then centrifuged at 4500*g* for 15 min to remove insoluble debris. The supernatant of the extracted inclusion bodies was applied to a nickel ion affinity chromatography (Ni-NTA) column to purify the recombinant protein. The purified recombinant protein was dialyzed stepwise at 4 °C with the following refolding buffers: refolding buffer 1 (4 M urea, 0.1 mM glutathione, 0.01 mM glutathione disulfide, 1 mM ethylene diamine tetraacetic acid, 5% (v/v) glycerol in PBS, pH=8.0) for 12 h; refolding buffer 2 (2 M urea, 0.1 mM glutathione, 0.01 mM glutathione disulfide, 1 mM ethylene diamine tetraacetic acid, 5% (v/v) glycerol, 0.15 M l-arginine in PBS, pH=8.0) for 12 h; refolding buffer 3 (1 M urea, 0.1 mM glutathione, 0.01 mM glutathione disulfide, 1 mM ethylene diamine tetraacetic acid, 5% (v/v) glycerol, in PBS, pH=8.0) for 12 h; and refolding buffer 4 (PBS, pH=8.0) for 12 h, twice. The proteins were subjected to sodium dodecyl sulfate polyacrylamide gel electrophoresis (SDS–PAGE), western blotting and protein size analyses.

To confirm P particle formation, the size distribution of the recombinant P particles was obtained by dynamic light scattering using a Zetasizer NANO ZS90 instrument (Malvern, Worcestershire, UK) with a fixed scattering angle of 90° at room temperature. Transmission electron microscopy (H-7650, Hitachi, Japan) was performed by dropping samples onto a carbon-formvar copper grid and negative staining with phosphotungstic acid. Transmission electron microscopy observations were conducted with an accelerating voltage of 80 kV, and images (50 k magnification) were obtained with a charge-coupled-device (CCD) camera system.

### SDS–PAGE and western blotting

SDS–PAGE was performed with 13.5% acrylamide gels in denaturing conditions. For the western blots, protein samples were mixed with 4 × SDS loading buffer (1 M Tris–HCl, pH 6.8, 8% SDS, 40% glycerol, 0.4 M dithiothreitol, 0.8% bromophenol blue) and boiled for 10 min at 98 °C before separation by 13.5% SDS–PAGE. The proteins were transferred onto nitrocellulose membranes (Whatman, Kent, UK). After the membranes were blocked with 3% nonfat milk at room temperature for 30 min, they were probed with an anti-His tag monoclonal antibody (Invitrogen, Carlsbad, CA, USA), followed by alkaline phosphatase-conjugated AffiniPure goat anti-mouse IgG (Beijing Dingguo, Inc., Beijing, China). Subsequently, the immunoreaction was detected with 5-bromo-4-chloro-3-indolylphosphate and nitroblue tetrazolium chloride solutions.

### Vaccination and challenge

In the first experiment, a total of 54 specific-pathogen-free BALB/c female mice (6-week-old) was randomly divided into nine groups and used for immunological evaluation. Mice (six animals per group) were subcutaneously immunized four times (2-week intervals) with 20 μg of the trivalent HA2-PP chimeric immunogen. As controls, mice were immunized with PBS; 20 μg of WT NoV P particles; 20 μg of HA2:90-105 peptides from the H1, H3 and B subtypes; 20 μg of HA2:90-105 peptides from the H1, H3 and B subtypes coupled to KLH; or H3 virus inoculated at 10^5^ 50% egg infective dose (EID_50_). The immunogens were administered in 100 μL of PBS mixed with an equal volume of a Freund's adjuvant (Beijing Dingguo, Inc.) per dose. To prevent nonspecific influenza virus infection, sera were collected from the mice immediately before each immunization for specific antibody detection. Serum collected at two weeks after each immunization was used to evaluate HA2-specific IgG titers by an enzyme-linked immunosorbent assay (ELISA).

In the second experiment, a total of 24 specific-pathogen-free BALB/c female mice (6-week-old) was randomly divided into four groups and used in a cross booster vaccination evaluation. Subtype-specific virus [A/Texas/JMM_30/2012(H3N2)] at 10^5^ EID_50_/0.1 mL was used as the booster for 20 μg of trivalent HA2-PP. Conversely, trivalent HA2-PP was used as the booster for subtype-specific virus as a control.

In the third experiment, a total of 12 specific-pathogen-free BALB/c female mice (6-week-old) was randomly divided into two groups and used to evaluate the outcome of immunization with virus challenge. The mice were subcutaneously immunized with 20 μg of the trivalent HA2-PP chimeric immunogen or with PBS as a control. Two to three weeks after boosting with the same immunogen, the mice were intranasally challenged with 10^7^ EID_50_/0.1 mL of A/Texas/JMM_30/2012(H3N2) virus. Before being infected with virus, the mice were anesthetized by the subcutaneous administration of a mixture of pentobarbital sodium (75 mg/kg body weight; Table 1).

### ELISA for serum antibody titer determination and antibody isotyping assay

HA2:76-130 peptides of the H1, H3 and B subtypes were used as antigens to detect HA2-specific antibodies. Ninety-six-well plates (Jet Biofil, Guangzhou, China) were coated with the abovementioned antigens at 5 μg/mL in PBS and kept overnight at 4 °C. The plates were blocked for 30 min at room temperature with 1% bovine serum albumin/PBS and then washed three times with PBS/0.05% Tween-20. Antisera from individual animals vaccinated with immunogens as mentioned above were serially diluted in 1% bovine serum albumin/PBS five times and added to the plates for incubation at 37 °C for 1.5 h. The plates were washed three times, and an anti-mouse horseradish peroxidase-conjugated antibody (Beijing Dingguo, Inc.) diluted at 1:2000 was added to the wells for incubation at 37 °C for 1 h. Subsequently, 3,3,5,5-tetramethyl benzidine solution was added to the wells for 20–30 min at room temperature. Optical density measurements were obtained at 450 nm. The highest reciprocal serum dilution that yielded absorbance >2-fold over the background value was determined as the ELISA end point titer.

For the antibody isotyping assay, anti-mouse IgG1, IgG2a, IgG2b, IgG3, IgM and IgA (Fc-specific; Sigma, St Louis, MO, USA) at a dilution of 1:1000 were added to the appropriate wells, and then, the plates were incubated for 1 h at room temperature. Anti-sheep IgG-alkaline phosphatase (Beijing Dingguo, Inc) diluted at 1:500 was used as the secondary antibody. p-Nitrophenyl phosphate substrate diluted in alkaline phosphatase buffer at 1 mg/mL was added to the wells and allowed to develop for 20–30 min at room temperature before optical density measurements were obtained at 405 nm.

### Hemagglutination inhibition (HAI) assay, microneutralization assay and detection of virus titer in lungs by reverse transcription-polymerase chain reaction (RT-PCR)

HAI assays were performed according to established protocols^25^ with modifications. In brief, each mouse serum sample was heat-inactivated at 56 °C for 0.5 h and then treated with receptor-destroying enzyme (Changchun BCHT Pharmaceutical Co., Ltd.) at 37 °C overnight according to the manufacturer's instructions. Subsequently, a 25-μL aliquot from each twofold serially diluted serum sample was incubated with 25 μL of virus (containing four HA units of influenza virus) at 37 °C for 1 h and then with 25 μL of 1% chicken red blood cells at 25 °C for 45 min. The HAI titer was defined as the reciprocal of the highest serum dilution that inhibited hemagglutination.

Microneutralization assay assays were performed according to established protocols with modifications.^26^ Up to 10 000 Madin–Darby canine kidney cells per well were grown in a 96-well plate in Dulbecco's modified Eagle's medium with 10% fetal bovine serum at 37 °C in 5% CO_2_. The cells were then washed with Hank's balanced salt solution (Sigma) three times until the cells adhered to the bottom of each well. Antisera from individual mice vaccinated with immunogens (introduced above) were serially diluted and reacted with a 200 × 50% tissue culture infective dose (TCID_50_) of different strains of IAV in virus medium (Dulbecco's modified Eagle's medium with 7.5% bovine serum albumin/PBS at volume ratio of 39:1). The mixture (antiserum and virus) was then placed into the appropriate wells and incubated at 37 °C in 5% CO_2_ for 2 h. Thereafter, the cells were washed with Hank's balanced salt solution three times and cultured in virus medium at 34 °C in 5% CO_2_. After 48 h, the supernatant from each well (100 μL) was collected for virus quantification by RT-PCR to evaluate the neutralizing activity of the sera. Lung tissue was also collected and tested with the real-time RT-PCR assay. Total RNA was prepared with the TIANamp RNA Kit for virus detection. RT-PCR was performed with a One-Step PrimeScript RT-PCR Kit (TaKaRa). The primers and the TaqMan probe used for RT-PCR are shown in Table 2.^27, 28^

### Homology remodeling

Homology models of the recombinant P particles were built with Discovery Studio version 2.1 software using the crystal structure of NoV TCH05 (PDB ID: 3SKB) as the template, which shares 98% sequence similarity with the recombinant P particles.

### Statistical analysis

The average value and s.d. for the level of immune responses within each group were calculated for comparison, and then, significant differences between results from the different groups were determined using Student's *t*-test with GraphPad Prism version 5.0 software (GraphPad Software Inc., San Diego, CA, USA).

## RESULTS

### Identification of highly conserved HA stem fragments from different virus subtypes

**Figure 1:**
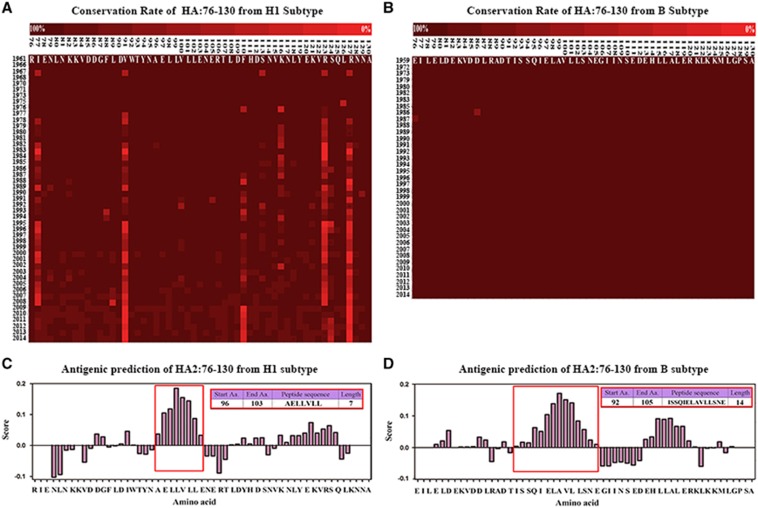
Conservation rate and B-cell epitope prediction of HA2:76-130 from subtypes H1, H3 and B. The sequences of HA2:76-130 from subtypes H1 (**A**) and B (**B**) are highlighted in colored progressive matrices based on the evaluated conservation rate. Conservation rates between 100% and 0% are indicated by dark to bright shades of red. B-cell epitopes were predicted in HA2:76-130 from the H1 subtype (**C**) and B subtype (**D**). Amino acid residues with scores higher than the threshold of 1.0 were considered potential B-cell epitopes. The framed sequences were ranked as the most likely B-cell epitopes.

Based on an alignment of HA stem fragments from H3 viruses in previous research and percent identity matrices of HA2:76-105 sequences comparing the H1, H3 and B subtypes, the conservation rate was 94±15.9467% for the H1 subtype (Figure 1A), 97.59±0.62% for the H3 subtype^18^ and 99.89±0.029293% for the B subtype (Figure 1B). In particular, the HA2:90-105 peptide sequence (H1: DIWTYNAELLVLLENE, H3: DLWSYNAELLVALENQ, B: DTISSQIELAVLLSNE), which could potentially be targeted by neutralizing antibodies with the significantly high conservation rate of 97±0.123240251% in the H1 subtype and 99.98±0.00283% in the B subtype, was predicted to contain potential B-cell epitopes (Figures 1C and D). Thus, constructs using a P particle expression vector were designed to express these sequences as described below.

### Development and characterization of a trivalent HA2-PP chimeric immunogen

**Figure 2:**
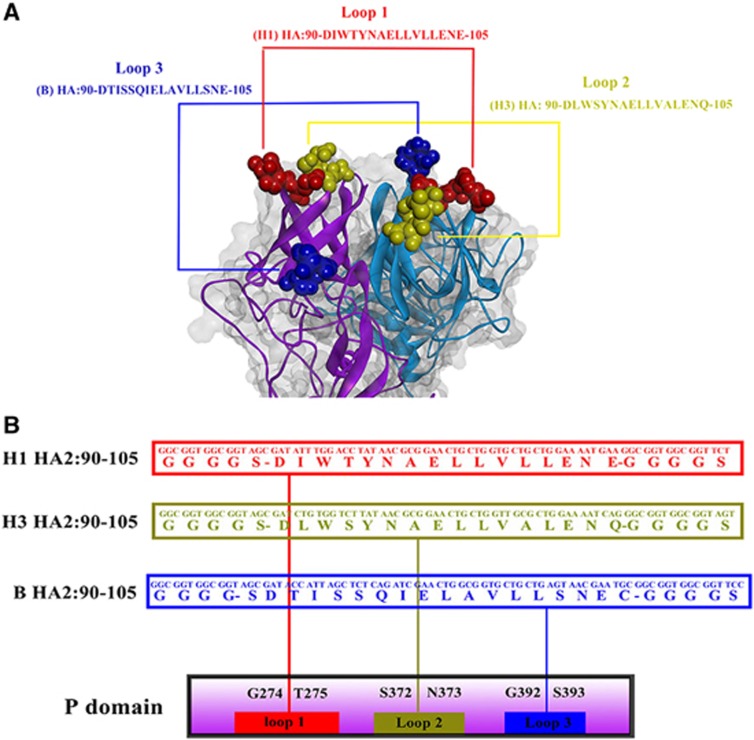
Expression constructs and homology remodeling of chimeric protrusion (P) particles. (**A**) Predicted loops of the HA2:90-105 region in NoV P particles are shown with the epitope fully exposed on the surface of the P particles. (**B**) The sequence DIWTYNAELLVLLENE from HA2 of the H1 virus (red) was inserted between the G274 and T275 residues in loop 1 of the NoV P domain via a GGGS linker. The sequence DLWSYNAELLVALENQ from HA2 of the H3 virus (yellow) was inserted between the S372 and N373 residues in loop 2 via a GGGS linker. The sequence DTISSQIELAVLLSNE from HA2 of the B virus (blue) was inserted between the G392 and S393 residues in loop 3 via a GGGS linker.

A recombinant P particle protein, trivalent HA2-PP, was developed by inserting one copy of each of the H1, H3 and B IAV consensus HA2:90-105 peptide sequences into the NoV P domain. The sequence DIWTYNAELLVLLENE from HA2 of H1 virus was inserted between the G274 and T275 residues of loop 1 of the NoV P domain via a GGGGS linker. The sequence DLWSYNAELLVALENQ from HA2 of H3 virus was inserted between the S372 and N373 residues of loop 2 via a GGGGS linker. The sequence DTISSQIELAVLLSNE from HA2 of B virus was inserted between the G392 and S393 residues of loop 3 via a GGGGS linker (Figure 2).

**Figure 3:**
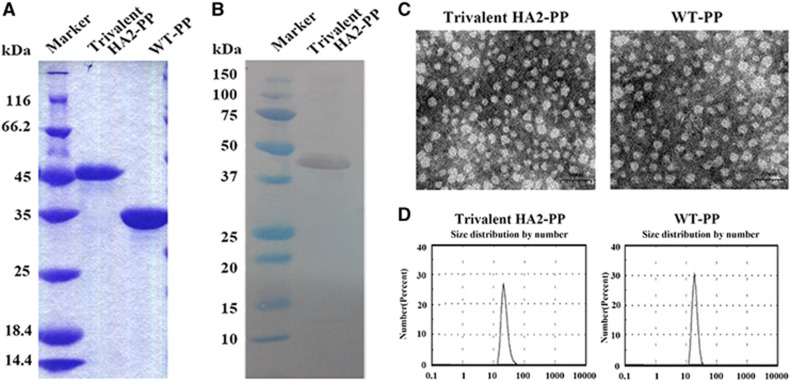
Production, purification and characterization of recombinant protrusion (P) particles. (**A**) Sodium dodecyl sulfate polyacrylamide gel electrophoresis (SDS–PAGE) analysis and (**B**) anti-His western blot analysis of chimeric P particles indicated that the size of trivalent HA2-PP was ~43 kDa, and the size of wild-type (WT) P particles was ~35 kDa. (**C**) Transmission electron microscopy (TEM) observations of recombinant P particles, scale bar, 50 nm. All of the P particles appeared to be globular according to TEM. (**D**) Size analysis of recombinant P particles indicated that they were all ~20 nm, suggesting that the purified NoV P domain could form 24-mer P particles *in vitro*.

The trivalent HA2-PP immunogen, with a molecular weight of 43.2 kDa as predicted using Gene Runner software, was prepared from *Escherichia coli* using Ni-NTA purification. All P particles were eluted with 300 mM imidazole (data not shown). The results from the SDS–PAGE analysis of the trivalent HA2-PP and WT P particles corresponded with their predicted molecular weight values (Figure 3A). Furthermore, western blot analysis confirmed that the trivalent HA2-PP contained the His tag (Figure 3B).

We collected P particles and characterized them by protein size analysis. The recombinant P particles were evenly distributed and appeared to be globular by transmission electron microscopy analysis (Figure 3C). In addition, the proteins were all ~20 nm in size (Figure 3D), suggesting that most of the recombinant P particle proteins could form 24-mers.

### HA2-specific antibody responses in mice vaccinated with the trivalent HA2-PP immunogen

**Figure 4:**
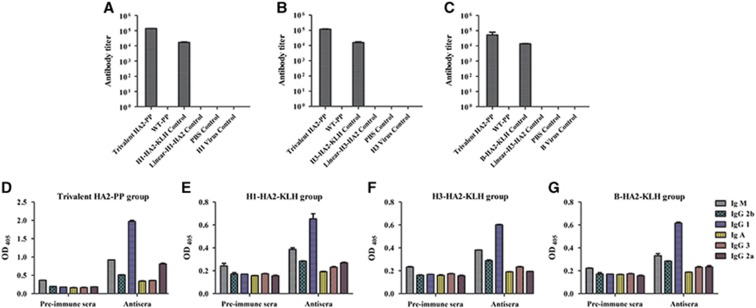
Detection of HA2-specific antibodies in mouse serum by enzyme-linked immunosorbent assay (ELISA) and an antibody isotyping assay. Antiserum titers were determined by ELISA with HA2:76-130 peptides from subtypes H1 (**A**), H3 (**B**) and B (**C**). The highest reciprocal serum dilution that yielded absorbance >2-fold over the background value was taken as the ELISA end point titer. Antibody isotyping was performed on sera from the trivalent HA2-PP group (**D**), the H1-HA2-KLH group (**E**), the H3-HA2-KLH group (**F**) and the B-HA2-KLH group (**G**). Titers and OD values are expressed as the mean±SD of six mice per group.

Anti-HA2 (H1, H3 and B subtypes) immune responses were monitored by determining the HA2:76-130-specific antibody titer in sera collected at two weeks after the fourth immunization. Mice vaccinated with either trivalent HA2-PP or a specific KLH immunogen via the subcutaneous route with Freund's adjuvant developed detectable HA2-specific serum titers (log_10_ ELISA titers in the range from 4–5). The trivalent HA2-PP immunogen was as much as 6.7 times more immunogenic than the KLH immunogen. Notably, no HA2-specific serum titer could be detected in the virus groups (Figures 4A–C). Subsequently, antiserum raised by either trivalent HA2-PP or KLH was analyzed by the antibody isotyping assay and was determined to be mainly IgG (Figures 4D–G).

**Figure 5:**
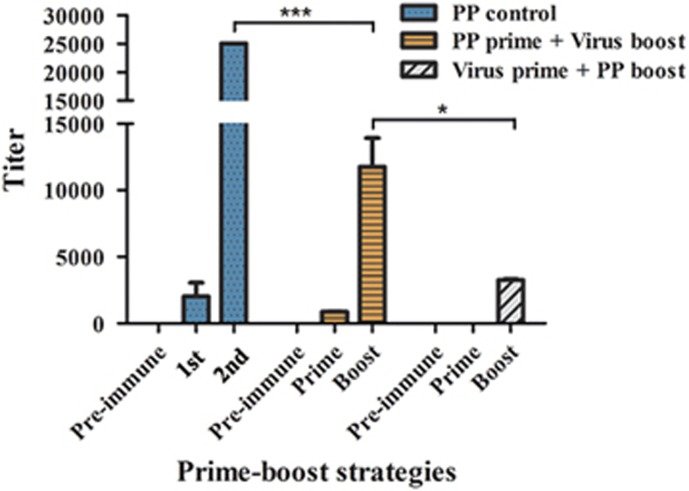
Detection of HA2-specific serum antibodies from cross booster vaccination experiments by enzyme-linked immunosorbent assay (ELISA). The A/Texas/JMM_30/2012(H3N2) virus was used as a booster for trivalent HA2-PP. As a control, trivalent HA2-PP was used as a booster for both the subtype-specific virus and trivalent HA2-PP. Antiserum titers were determined by ELISA with HA2:76-130 peptide of H3 subtype. The highest reciprocal serum dilution that yielded absorbance >2-fold over the background value was taken as the ELISA end point titer. Titers are expressed as the mean±SD of six mice per group. *P<0.05, ***P<0.0005.

In cross booster vaccination experiments, based on the results obtained from the first experiment, a subtype-specific virus [A/Texas/JMM_30/2012(H3N2)] was used as the booster for trivalent HA2-PP. Conversely, trivalent HA2-PP was used as the booster for the subtype-specific virus as a control. The results showed that trivalent HA2-PP, when given with the subtype-specific virus booster, was highly immunogenic, with log_10_ ELISA titers in the range from 3.98–4.14. In addition, the subtype-specific virus with the trivalent HA2-PP booster as a control was weakly immunogenic, with log_10_ ELISA titers of ~3.5 after the boost, and no specific titer was detected after the prime immunization (Figure 5).

### Broadly neutralizing antisera elicited by trivalent HA2-PP

The HAI activity of antiserum induced by either trivalent HA2-PP or KLH did not significantly differ from that of the pre-immune serum as shown in Table 3. However, mice intranasally vaccinated with virus developed significant HAI titers.

In the microneutralization assay, antisera from both the trivalent HA2-PP and KLH groups showed strong neutralizing activity against two clades of H3 virus, with high half maximal inhibitory dilution (ID_50_) values (80–1378; *P*<0.05) and neutralizing activity against B virus with lower ID_50_ values (45–156.8; *P*<0.05; Table 4).

### Protective efficacy of trivalent HA2-PP against challenge with H3 subtype IAV

**Figure 6:**
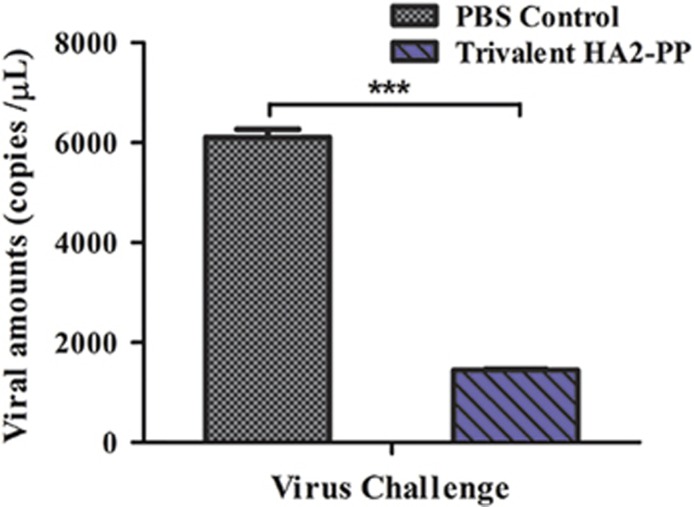
Viral copy number in lungs on day 3 post-challenge. Mice from each challenge group were killed three days post-challenge, and the amount of virus in the lungs was determined by real-time PCR. Values are expressed as the mean log10 viral copies/μL±SD of six mice per challenge group. ***P<0.0001, trivalent HA2-PP challenged group compared with phosphate-buffered saline (PBS) control.

Given the strong serum neutralizing activity observed against two clades of H3 virus with high ID_50_, viral replication was also monitored in the lungs of mice challenged with 10^7^ EID_50_/0.1 mL of the A/Texas/JMM_30/2012(H3N2) strain. Viruses in the lungs of PBS control mice replicated to 3.774–3.797 log_10_ copies/μL, whereas those in mice immunized with trivalent HA2-PP replicated to 3.153–3.165 log_10_ copies/μL. Thus, the average amount of virus in the lungs of trivalent HA2-PP-vaccinated mice was significantly lower (*P*<0.001) after viral challenge than that in the PBS control group (Figure 6).

## DISCUSSION

IAV outbreaks are among the largest disease epidemics that influence both human health and productivity. Although seasonal vaccines help to curb the spread of infection, the mutability of IAV sequences under pressure from neutralizing antibodies hampers the development of universal influenza vaccines.^1^ Based on the knowledge that a low-pH-induced conformational change in the HA stem is required for membrane fusion and viral escape from neutralization,^29^ we focused on the stem fragment of HA2 encompassing the entire α-helix. First, a large data set (17 524 sequences for the H1 subtype, 10 763 sequences for the H3 subtype and 4022 sequences for the B subtype) was analyzed to identify conserved epitopes in this stem fragment. Consistent with results from previous studies,^30^ the stem fragment was confirmed to be more conserved than the highly variable globular head domain. Therefore, a stem fragment containing an epitope targeted by broadly neutralizing antibodies may be a potential candidate for the development of a broadly protective vaccine.

As a hapten, HA2:90-105 is poorly immunogenic, and thus, a carrier is important for improving its immunogenicity. The NoV P particle, which has a three-ring structure domain suitable for the insertion and presentation of the epitope, has been demonstrated to be an ideal platform for presenting antigens.^31^ Moreover, P particles are able to self-assemble into 24-mers, which are conducive to presenting multiple epitopes and inducing high levels of specific antibodies. Considering the known immunogenicity and protective efficacy of HA2:90-105 from the H3 subtype, in this study, a trivalent chimeric HA2-PP protein was constructed using a P particle expression vector with one copy each of the IAV H1, H3 and B subtype consensus HA2:90-105 peptide sequences inserted into loops 1, 2 and 3, respectively. The current results demonstrated that the recombinant P particle-based protein was successfully prepared. After immunization of mice, as expected, this chimeric P particle, compared with WT P particles, induced a strong and specific IgG antibody response against subtype-specific HA2 epitopes. Notably, no HA2-specific binding activity was detectable in the virus control. This result is consistent with a previous determination that the HA2:90-105 region is exposed for a limited period of time during membrane fusion.^18^ In cross booster vaccination experiments, the serum antibody titer induced by trivalent HA2-PP could be boosted by a subtype-specific virus but not vice versa. Therefore, the induction of high-titer antibodies to the stem fragment strongly suggests that the identified epitopes could be applied in a vaccine candidate to specifically boost low levels of preexisting stem-directed cross-reactive antibodies in the human population.

Antisera from both the trivalent HA2-PP and KLH groups showed *in vitro* neutralizing activity against H3 virus and B virus but not H1 virus. Although the trivalent HA2-PP was more immunogenic, compared with the KLH group, it induced antisera with weaker neutralizing activity. These different results may be reconciled by one of two explanations. First, because neutralizing activity was not detected against the H1 virus, the HA2:90-105 epitope of the H1 subtype may not be exposed for a sufficient period of time during membrane fusion. Second, the weaker neutralizing activity of serum from the trivalent HA2-PP group may indicate that the conformational flexibility is not the same, even though the same epitope is presented by both immunogens. Interestingly, specific antibody titers to the trivalent HA2-PP lasted as long as 1 year (data not shown), strongly suggesting that HA2-PP may be applied as a vaccine candidate to provide long-term protection. Similarly to previous reports,^10, 11^ the trivalent HA2-PP immunogen in this study expresses the trimeric HA stem fragment in loops 1, 2 and 3 in such a manner as to mimic the natural HA trimer, thereby increasing its immunogenicity.

In terms of protective efficacy of the chimeric vaccine, we identified enhanced neutralizing activity against the H3 subtype virus, which may represent a correlate of protection in mice against H3 virus challenge. Vaccination with the trivalent HA2-PP but not with the PBS control significantly reduced virus titers in the lung after challenge. Further studies are needed to assess the immune protection provided by this vaccine against other virus subtypes. The trivalent HA2-PP was found to be immunogenic when subcutaneously administered to mice with Freund's adjuvant, and it provided protective antibody responses as assessed *in vitro* and *in vivo* against H3 and B subtypes of IAV. Evaluating other possible innate and/or adaptive protection mechanisms induced by the HA2 stem in a mouse model would be worthwhile in future work. With further improvement of the trivalent HA2-PP immunogen construct and optimization of the dose and vaccination strategy, this vaccine candidate has the potential to be applied in the clinic as one component of a broadly protective vaccine.

## Figures and Tables

**Table 1:** Mouse immunization groups and challenge strains

**Experiment**	**Group**	**Adjuvant**	**Dose**	**Immunization scheme**		**Challenge strain**
				**Prime**	**Boost**	
Immunological experiments	Trivalent HA2-PP	Yes	20 μg	Four immunizations		—
	WT-PP	Yes	20 μg	Four immunizations		—
	PBS control	Yes	—	Four immunizations		—
	Linear-H1-HA2 control	Yes	20 μg	Four immunizations		—
	Linear-H3-HA2 control	Yes	20 μg	Four immunizations		—
	Linear-B-HA2 control	Yes	20 μg	Four immunizations		—
	H1-HA2-KLH control	Yes	20 μg	Four immunizations		—
	H3-HA2-KLH control	Yes	20 μg	Four immunizations		—
	B-HA2-KLH control	Yes	20 μg	Four immunizations		—
	H1 virus control^a^	No	10^5^ EID_50_	Three immunizations		—
	H3 virus control^b^	No	10^5^ EID_50_	Three immunizations		
	B virus control^c^	No	10^5^ EID_50_	Three immunizations		
Cross booster vaccination experiments	Trivalent HA2-PP (prime/boost)	Yes	20 μg	Trivalent HA2-PP	Trivalent HA2-PP	—
	Virus (prime/boost)^b^	No	10^5^ EID_50_	Virus	Virus	—
	Trivalent HA2-PP/ virus	Yes	20 μg/10^5^EID_50_	Trivalent HA2-PP	Virus	—
	Virus/ trivalent HA2-PP	Yes	10^5^EID_50_/20 μg	Virus	Trivalent HA2-PP	—
Virus challenge experiments	Trivalent HA2-PP	Yes	20 μg	Three immunizations		H3N2^b^
	PBS	Yes	—	Three immunizations		H3N2^b^

Abbreviations: Egg infective dose, EID_50_; keyhole limpet hemocyanin, KLH; phosphate-buffered saline, PBS; wild-type P particles, WT-PP.

a A/17/California/2009/38(H1N1) virus.

b A/Texas/JMM_30/2012(H3N2) virus.

c B/56/Brisbane/60/80 virus.

**Table 2:** Sequences of the primers and probes used for the RT-PCR assay

**Subtype**	**Strain**	**Target gene**	**Nucleotides**	**Primer sequences (5′–3′)**	
H1	A/17/California/2009/38	HA	677–698	Sense:	AGTTCAAGCCGGAAATAGCAAT
			823–845	Antisense:	ATACCAGATCCAGCATTTCTTTC
			704–731	Probe:	FAM-CCAAAGTGAGGGATCAAGAAGGGAGAAT-BHQ1
H3	A/17/Perth/16/2009	HA	296–314	Sense:	CAGTGTGATGGCTTCCAAA
	A/Wisconsin/67/2005		375–392	Antisense:	AGGCATAATCCGGCACAT
	A/Texas/JMM_30/2012		340–365	Probe:	HEX-ACGCAGCAAAGCCTACAGCAACTGTT-BHQ1
B	B/56/Brisbane/60/80	NP	219–237	Sense:	CAACCACAAGCAGTGAAGC
	B/60/Massachusetts/20/2012		340–318	Antisense:	CATCATCTGGTTGTAGAATTCAC
			265–294	Probe:	CY5-ACGCTCTTCTTTATCTCTGTCGGGGTTTGT-BHQ2

Abbreviations: hemagglutinin, HA; nuclear protein, NP.

**Table 3:** HAI titer of antisera against different viruses

**Antiserum groups**	**Virus**					
	**H1 subtype**	**H3 subtype**			**B subtype**	
	**A/17/California/2009/38**	**A/17/Perth/16/2009**	**A/Wisconsin/67/2005**	**A/Texas/JMM_30/2012**	**B/56/Brisbane/60/80**	**B/60/Massachusetts/20/2012**
Trivalent HA2-PP	32–64^N^ a	32^N^	64^N^	64^N^	16–32^N^	32–64^N^
H3-HA2-KLH	32–64^N^	16–32^N^	32–64^N^	64^N^	16–32^N^	16–32^N^
Virus	128* b	128*	128*	128–256*	256*	256*

Abbreviations: hemagglutination inhibition, HAI; keyhole limpet hemocyanin, KLH.

a No significant difference in HAI activity was detected between antiserum and pre-immune serum.

b Significant difference in HAI activity was detected between antiserum and pre-immune serum from mice given virus immunogens (*P<0.05).

**Table 4:** Neutralizing activity (ID_50_) of antisera against different viruses

**Antiserum group**	**Virus**				
	**H1 subtype**	**H3 subtype**		**B subtype**	
	**A/17/California/2009/38**	**A/17/Perth/16/2009**	**A/Texas/JMM_30/2012**	**B/56/Brisbane/60/80**	**B/60/Massachusetts/20/2012**
Trivalent HA2-PP	—a	656*^b^	187*	—	45*
WT-PP	—	—	—	—	—
H1-HA2-KLH	—	—	—	—	—
H3-HA2-KLH	—	1378**	564.57*	87.9*	156.8*
B-HA2-KLH	—	80*	—	50*	105*

Abbreviations: keyhole limpet hemocyanin, KLH; microneutralization assay, MN.

a No significant difference in MN activity was detected between antiserum and pre-immune serum.

b Significant difference in MN activity was detected between antiserum and pre-immune serum (*P<0.05, **P<0.005, ***P<0.0005).
